# Double-Row Suture Anchor Fixation for Inferior Pole Patella Fracture: A Novel Technique

**DOI:** 10.7759/cureus.105610

**Published:** 2026-03-21

**Authors:** Qing Hang Tan, Foong Wei Sheng, Dalun Leong, Ing How Moo

**Affiliations:** 1 Department of Orthopaedic Surgery, Changi General Hospital, Singapore, SGP

**Keywords:** case report, double-row technique, inferior pole patella fractures, novel technique, suture anchor fixation

## Abstract

Inferior pole patella fractures are challenging to treat due to small, comminuted fragments and the high tensile forces generated by the extensor mechanism. Conventional surgical fixation techniques are associated with implant irritation, migration, hardware failure, and the need for secondary procedures.

This case report describes a novel double-row suture anchor fixation technique with suture tape augmentation for treating an inferior pole patella fracture. An 80-year-old patient with a displaced comminuted inferior pole fracture underwent surgical fixation using obliquely inserted double-row suture anchors and a supplementary figure-of-eight tension-band construct. Early union, good reduction, and restoration of knee range of motion were achieved without complications.

This technique provided stable fixation, minimized soft-tissue irritation, and required minimal intraoperative fluoroscopy. The described method represents a reliable and reproducible alternative to conventional metallic fixation, particularly in osteoporotic bone.

## Introduction

Inferior pole patella fractures account for 5% of all patella fractures [1] and frequently require surgical intervention to restore extensor mechanism continuity. These types of fractures are technically challenging due to the small, often comminuted distal fragments and poor bone quality. Both of which compromise fixation strength, especially in elderly patients [2]. Traditional fixation methods, including tension-band wiring, transosseous sutures, and partial patellectomy, have been associated with complications such as implant migration, irritation, hardware prominence, and patella baja, leading to high rates of revision surgery [3 - 5].

Recent interest in non-metallic fixation materials has shown potential benefits. A systematic review on non-metallic implants for patella fixation reported an overall success rate of 90%, mainly due to implant removal from chronic irritation and loss of reduction [6]. We propose a novel double-row suture anchor fixation technique utilizing fiber tape as a tension-band device designed to enhance fixation stability, avoid patellar tendon violation, minimize implant-related issues, and enable early effective rehabilitation. A single clinical case is presented to demonstrate the feasibility and early clinical outcome of this method. Written informed consent was taken from the patient for both surgery and publication. 

## Case presentation

**Figure 1:**
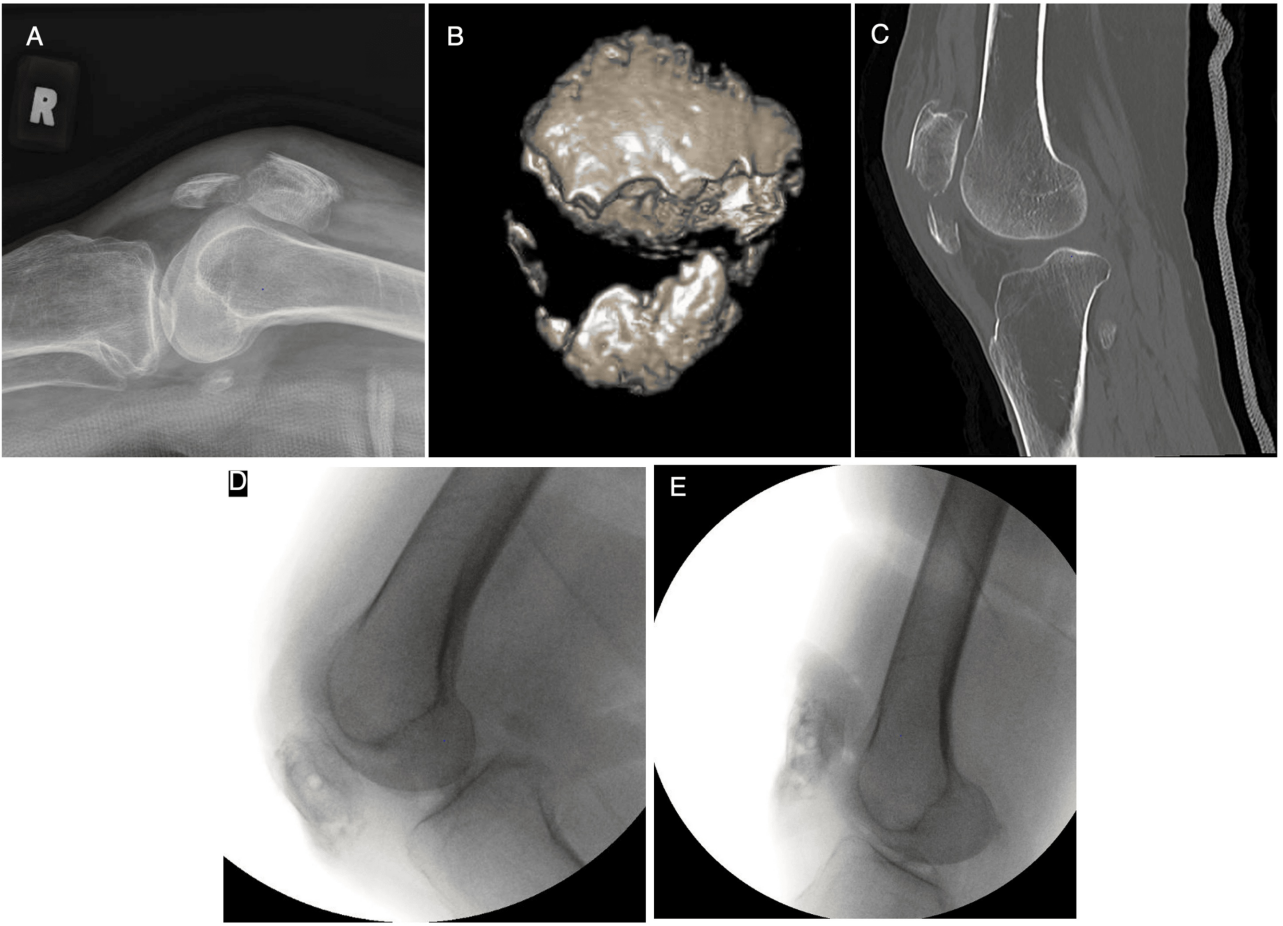
Pre-operative X-ray and computed tomography of right knee with 3D reconstruction A: Pre-operative plain radiograph of the right knee shows a fracture of the inferior pole of the patella with obvious displacement of the fractured end. B & C: Computed tomographic knee imaging with 3D reconstruction showing the comminuted small bone fragments in the inferior pole of the patella. D & E: Intraoperative imaging of the right knee showing good fracture reduction and stability with 90 degrees of flexion and full extension of the knee.

An 80-year-old patient presented to the hospital with right knee pain following a fall. Plain radiographs of the knee (Figure 1A) and computed tomographic knee imaging with 3D reconstruction (Figures 1B, 1C) revealed a displaced comminuted inferior pole patella fracture (AO 34-A1). She was medically fit for surgery and underwent a novel double-row suture anchor fixation technique. Two radiographic images of fracture fixation were taken intraoperatively (Figures 1D, 1E) with an operative time of 58 minutes. 

Surgical technique

**Figure 2:**
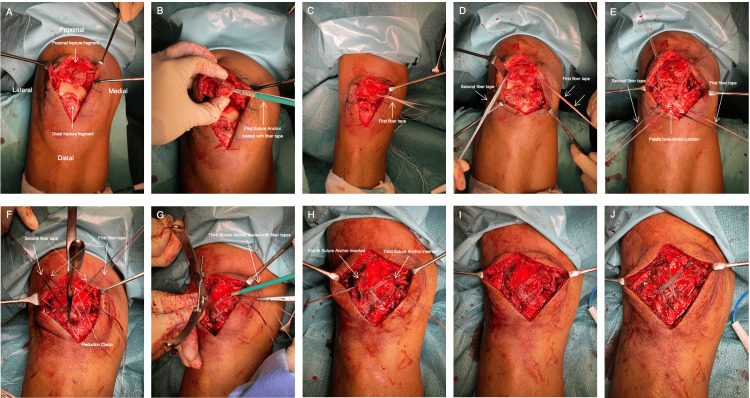
Step by step technique of double row suture anchor fixation for inferior pole patella fracture A: Fracture site exposed; B, C & D: Two suture anchors, each loaded with a fiber tape, were inserted obliquely on either side of the proximal fragment outside the fracture; E: A trocar needle was used to pass the four limbs of the suture tapes through the patellar tendon at the edge of the inferior pole of the patella as shown; F: The fracture was anatomically reduced with a patellar reduction clamp; G & H: One fiber tape from each side was loaded onto the suture anchor and inserted on opposite side of the quadriceps tendon at the proximal fragment, at approximately the 2 o’clock and 10 o’clock positions; I: The fracture fixation completed after removal of remnant fiber tape; J: Another fiber tape was folded in half to double the stack and used to pass under the patella and quadriceps tendon in a figure-eight fashion to serve as a tension-band construct.

The surgery was performed with the patient in the supine position, and a radiolucent bolster was positioned below the thigh to maintain slight knee flexion. A standard midline incision was made, extending from the superior to the inferior pole of the patella, without exposing the patellar tendon. The hematoma was evacuated, and fracture edges were debrided to clearly define the fracture margins (Figure 2A).

**Figure 3:**
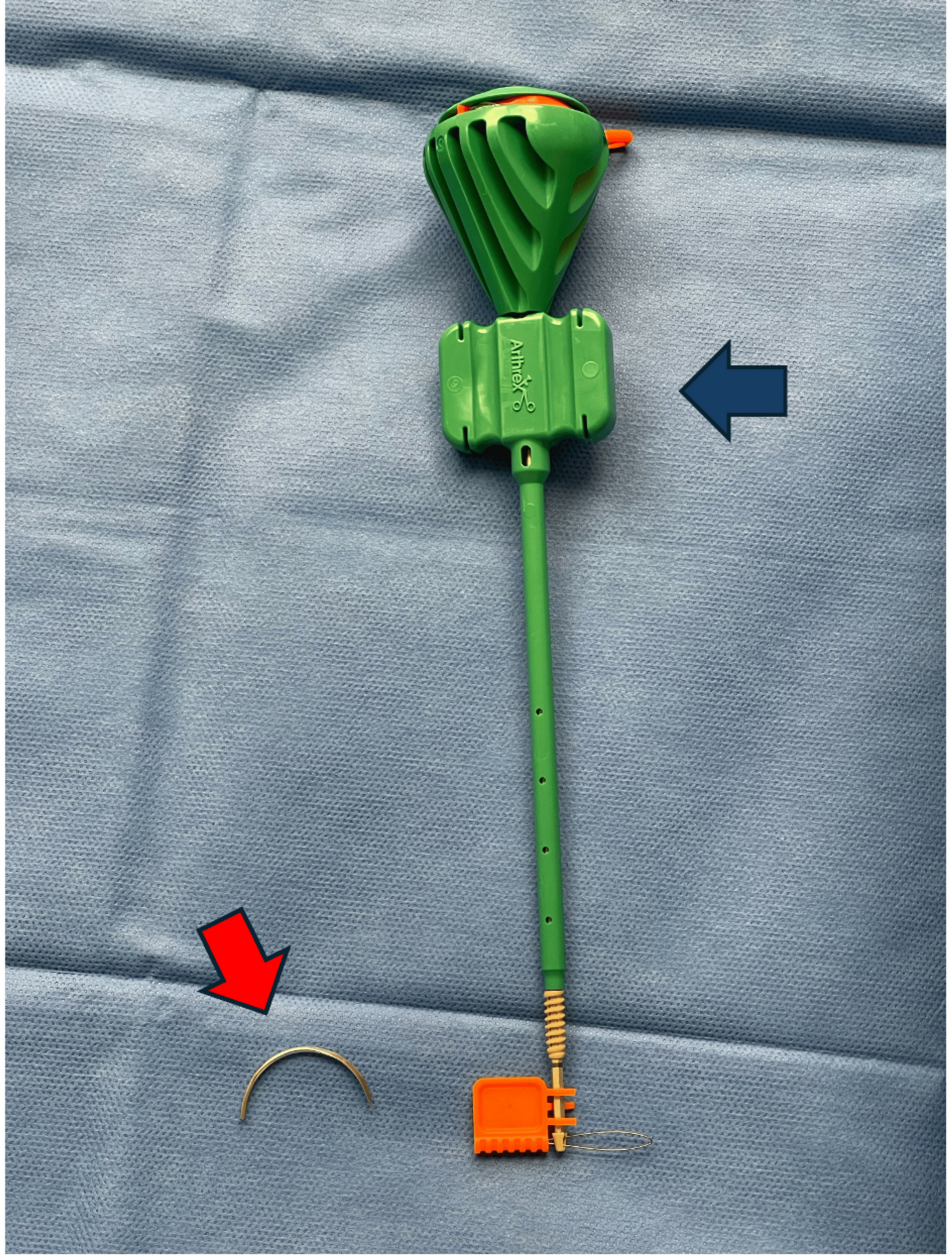
Size 4 trocar needle and polyetheretherketone (PEEK) Knotless Swivelock Suture Anchor (4.75 mm × 19.1 mm) (Arthrex Inc., Naples, FL, USA) The red arrow indicates size 4 trocar needles, whereas the blue arrow indicates the PEEK Knotless Swivelock Suture Anchor.

Two polyetheretherketone (PEEK) knotless SwiveLock suture anchors (4.75 mm × 19.1 mm) (Arthrex Inc., Naples, FL, USA) (Figure 3), each loaded with fiber tape, were inserted at least 45 degrees obliquely relative to the tension force axis on either side of the proximal fragment outside the fracture (Figures 2B, 2C, 2D). A size-4 trocar needle (Figure 3) was used to pass the four limbs of the fiber tapes through the patellar tendon at the edge of the inferior pole of the patella. Fiber tapes from each side of the suture anchors had two limbs, one limb passes through the patellar tendon at the ipsilateral side of the edge of the distal fragment, while another limb passes through the contralateral side (Figure 2E). With the knee in full extension, the fracture was anatomically reduced and compressed using a patellar reduction clamp (Figure 2F). The slack in the suture tape was then removed.

One tape from each side of the suture anchor was then loaded onto an additional pair of PEEK Knotless Swivelock Suture Anchors (4.75 mm × 19.11 mm) and inserted on the opposite side of the quadriceps tendon into the proximal fragment at approximately the 2 o’clock and 10 o’clock positions (Figures 2G, 2H). Upon advancing these suture anchors into the bone, ensuring the fiber tape was kept in tension, and the anchor was flushed or slightly deeper to the bone surface, this further compressed the fracture site and completed the double-row suture anchor construct (Figure 2I). 

**Figure 4:**
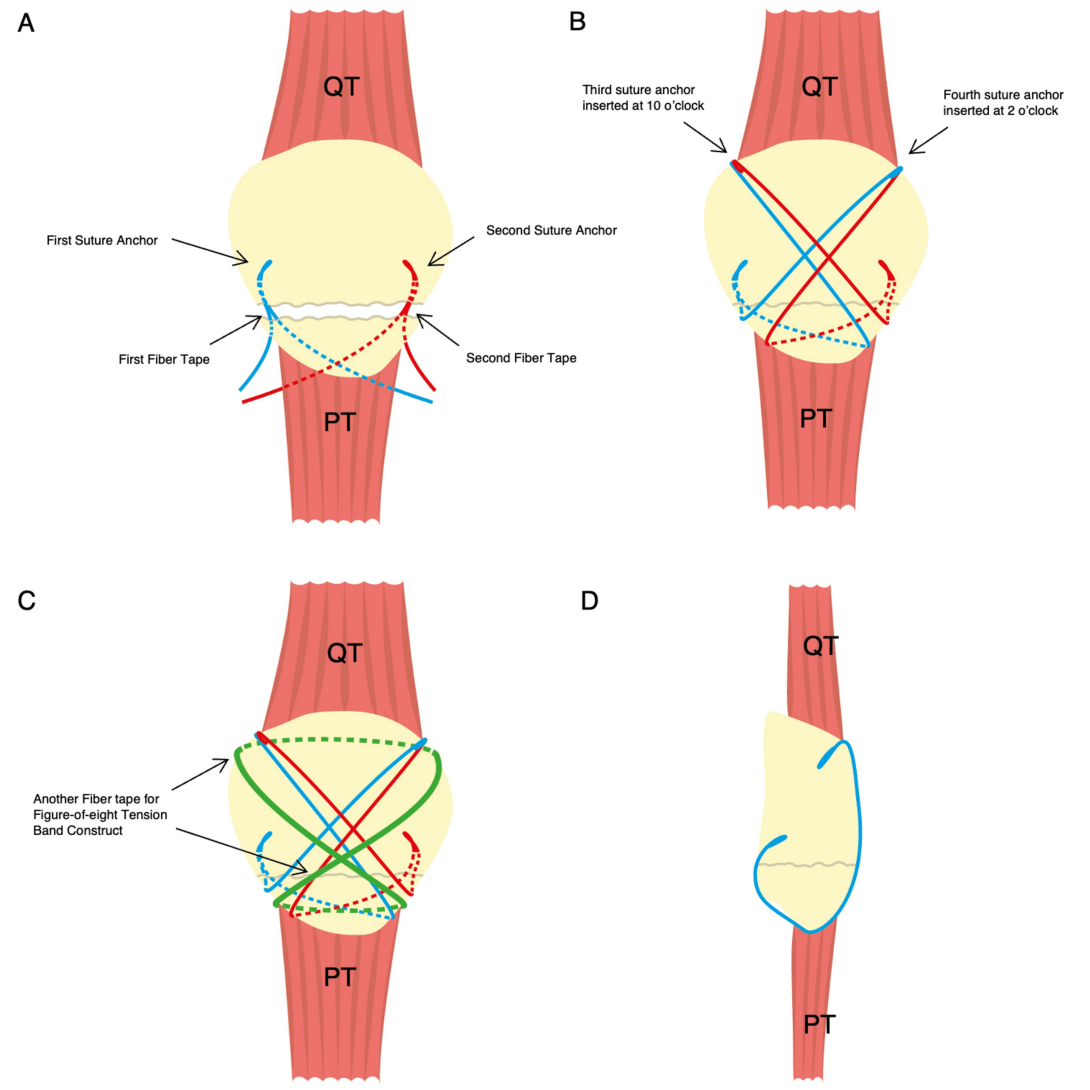
The double-row suture anchor technique used to treat an inferior pole patella fracture A: Two suture anchors loaded with fiber tape were inserted obliquely on either side of the proximal fragments, and a trocar needle was used to pass all the sutures through the patella tendon (PT) at the edges of either side of the inferior fragment. B: The fracture was reduced, and sutures were then brought to the opposite side of the quadriceps tendon (QT) of the proximal fragment and secured with a suture anchor at the 10 and 2 o’clock positions. C: The fracture fixation was augmented with fiber tape in a figure-of-eight fashion to create a tension-band construct. D: Sagittal view of inferior patella pole fixation. This is a freehand sketch of the double-row suture anchor technique used to treat inferior pole patella fracture, created using Adobe Illustrator (Adobe Inc., San Jose, CA, USA). Image credit: Dr. Lee Bing Qian.

A 36-inch Arthrex fiber tape (Arthrex Inc.) was folded in half to double stack and passed under the patella and quadriceps tendons in a figure-eight fashion, serving as a tension-band construct (Figure 2J). Stability of the reduction was assessed by cycling the knee through 20 repetitions. Intraoperative imaging of the knee was performed twice: once with the knee in full extension and once at 90 degrees of flexion. A diagrammatic illustration of the surgical technique is shown in Figure 4.

Postoperative course 

The patient was placed in a knee brace with controlled mobilization, active knee range of motion of 0 to 30 degrees for the first four weeks, with 30-degree increments every two weeks thereafter. Immediate isometric exercises and straight leg raises were allowed. Weight bearing with the brace locked in extension was initiated for four weeks. In total, the knee brace was maintained for eight to 12 weeks. 

**Figure 5:**
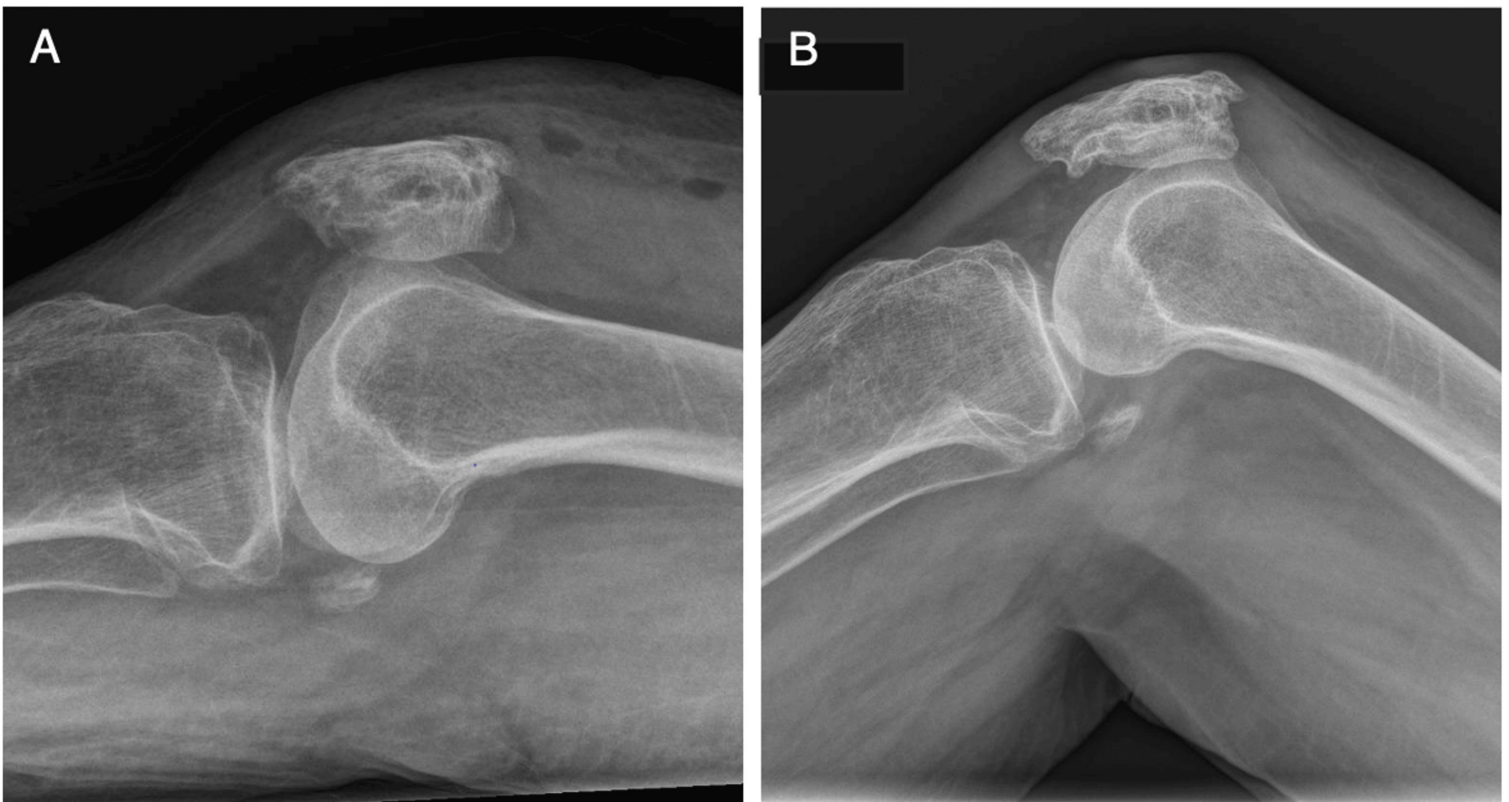
Postoperative X-ray of the right knee A: Postoperative plain radiograph of the right knee at the three-month follow-up, showing good bony union without loss of reduction. B: Postoperative plain radiograph of the right knee at the one-year follow-up, showing good bony union with no loss of articular congruity.

Radiographs at three months (Figure 5A) showed complete bony union without displacement. At 1-year follow-up (Figure 5B), she demonstrated a knee range of motion from 0° to 120°, a Visual Analog Scale score of 1 [7], and a Kujala score of 74/100 [8]. No complications such as implant irritation, loss of reduction, or need for revision surgery were observed (Table 1).

**Table 1:** Clinical parameters of the case

Parameters	Values
Operative time	58 minutes
Intraoperative fluoroscopy images	Two images
Postoperative complications	Nil
Fracture union	Three months
Revision surgery	Nil
Range of motion of the knee one year postoperatively	0 – 120 degrees
Visual Analog Scale score post operation [7]	1
Kujala score one year postoperatively [8]	74/100

## Discussion

Inferior pole fractures of the patella remain surgically demanding due to small, often comminuted fragments and poor bone quality encountered in elderly patients [9]. Conventional fixation techniques such as tension-band wiring, transosseous sutures, and partial patellectomy [5] carry several limitations, including hardware-related complications such as implant migration, patella tendon violation, and fixation failure, resulting in a high rate of revision surgery [10]. These drawbacks are accentuated in osteoporotic bone, where rigid metallic fixation may fail due to limited bone stock. In contrast, suture-based fixation offers biologic advantages but may be limited by insufficient strength when using single-row constructs [4]. 

Our novel double-row suture anchor technique with suture tape tension-band augmentation, described in this study, aims to address these biomechanical and clinical shortcomings. The double-row construct distributes tension forces over a broader surface area between the proximal and distal rows, reducing stress concentration at the bone-tendon interface, while the supplementary figure-of-eight tension band further converts tensile forces into compressive forces across the fracture plane. Forty-five-degree oblique anchor placement maximizes resistance to tensile forces generated by the quadriceps and reduces the risk of anchor pullout. 

Compared with transosseous repair, this approach avoids patellar tendon violation, reduces invasiveness, and prevents patella baja [5, 11]. Unlike metallic implants, suture tape causes minimal soft-tissue irritation and eliminates the need for hardware removal. The technique also facilitates fixation with the knee in extension, which neutralizes the high tensile force originating from the patella and quadriceps tendon, making anatomic reduction easier and reducing the need for fluoroscopy. The author managed to take fewer than three intraoperative images in the case illustration. Owing to these advantages, the operative timing can be shortened to within 60 minutes without compromising fracture fixation. The technique facilitates smaller incisions with easy fracture reduction and extra-articular suture anchor placement. 

Suture tape typically causes less soft tissue irritation than metallic wires due to their softer, thinner, and flatter profile, which reduces pressure on the soft tissues and lowers the risk of prominence and irritation by around 30% to 60%, with a reported revision surgery rate of up to 37% [12-14]. Biomechanical studies have demonstrated that suture tape has comparable biomechanical properties, such as load to failure, stiffness, and resistance to cyclic displacement at the bone-tendon interface, as compared to metallic fixation [15-18]. 

Early clinical outcomes in this case, union at three months, restoration of full knee motion, and absence of complications, support the potential advantages of this technique. However, limitations include the single-case design and the absence of biomechanical validation. Larger cohort studies and cadaveric testing are underway to further evaluate fixation strength, anchor orientation, and comparative outcomes versus traditional methods.

## Conclusions

This novel double-row suture-anchor technique effectively addresses the common challenges associated with conventional methods for inferior pole patella fractures. We believe this technique provides a reliable, reproducible, and biologically favorable fixation method to achieve satisfactory fracture reduction and firm fixation, facilitating early rehabilitation as well as mitigating the rate of revision surgery. Additionally, the included case with this novel technique achieved fracture union and a satisfactory range of motion without implant-related complications. Although our initial findings are promising, more comprehensive studies are required to further validate the clinical evidence as well as the relevance of this technique.

## References

[1] Yang X, Wu Q, Lai CH, Wang X (2017). Management of displaced inferior patellar pole fractures with modified tension band technique combined with cable cerclage using Cable Grip System. Injury.

[2] S. L. J. Yuen and M H. Chau (2025). A review of a new technique for comminuted inferior pole patellar fracture—modified non-metallic separate vertical wiring (NM-SVW). J Orthop Trauma Rehabil.

[3] Chang CH, Shih CA, Kuan FC, Hong CK, Su WR, Hsu KL (2023). Surgical treatment of inferior pole fractures of the patella: a systematic review. J Exp Orthop.

[4] Chang CH, Chuang HC, Su WR, Kuan FC, Hong CK, Hsu KL (2021). Fracture of the inferior pole of the patella: tension band wiring versus transosseous reattachment. J Orthop Surg Res.

[5] Hung LK, Lee SY, Leung KS, Chan KM, Nicholl LA (1993). Partial patellectomy for patellar fracture: tension band wiring and early mobilization. J Orthop Trauma.

[6] Camarda L, Morello S, Balistreri F, D'Arienzo A, D'Arienzo M (2016). Non-metallic implant for patellar fracture fixation: a systematic review. Injury.

[7] Huskisson EC (1974). Measurement of pain. Lancet.

[8] Kujala UM, Jaakkola LH, Koskinen SK, Taimela S, Hurme M, Nelimarkka O (1993). Scoring of patellofemoral disorders. Arthroscopy.

[9] Pu S, Chen Y, Liang J, Xu Y, Zhao Y (2022). Treatment of inferior pole fracture of the patella with tension-free external immobilization. BMC Surg.

[10] Kadar A, Sherman H, Glazer Y, Katz E, Steinberg EL (2015). Predictors for nonunion, reoperation and infection after surgical fixation of patellar fracture. J Orthop Sci.

[11] O'Donnell R, Lemme NJ, Marcaccio S, Walsh DF, Shah KN, Owens BD, DeFroda SF (2021). Suture anchor versus transosseous tunnel repair for inferior pole patellar fractures treated with partial patellectomy and tendon advancement: a biomechanical study. Orthop J Sports Med.

[12] Böstman O, Kiviluoto O, Santavirta S, Nirhamo J, Wilppula E (1983). Fractures of the patella treated by operation. Arch Orthop Trauma Surg (1978).

[13] Bushnell BD, Byram IR, Weinhold PS, Creighton RA (2006). The use of suture anchors in repair of the ruptured patellar tendon: a biomechanical study. Am J Sports Med.

[14] Hoshino CM, Tran W, Tiberi JV, Black MH, Li BH, Gold SM, Navarro RA (2013). Complications following tension-band fixation of patellar fractures with cannulated screws compared with Kirschner wires. J Bone Joint Surg Am.

[15] Piatti M, Gorla M, Alberio F (2023). Comparison of all-suture anchors with metallic anchors in arthroscopic cuff repair: structural and functional properties and clinical suitability. J Orthop.

[16] Denard PJ, Burkhart SS (2013). Double-row suture-bridging arthroscopic rotator cuff repair. Oper Tech Orthop.

[17] Ravalin RV, Mazzocca AD, Grady-Benson JC, Nissen CW, Adams DJ (2002). Biomechanical comparison of patellar tendon repairs in a cadaver model: an evaluation of gap formation at the repair site with cyclic loading. Am J Sports Med.

[18] Mall NA, Lee AS, Chahal J, Van Thiel GS, Romeo AA, Verma NN, Cole BJ (2013). Transosseous-equivalent rotator cuff repair: a systematic review on the biomechanical importance of tying the medial row. Arthroscopy.

